# Penetration testing of a quantum key distribution system as a black box

**DOI:** 10.1093/nsr/nwag174

**Published:** 2026-03-19

**Authors:** Anqi Huang, Qingquan Peng, Junxuan Liu, Xialong Yuan, Cheng Peng, Zhengyuan Yang, Hansen Wu, Zihao Chen, Guangfu Sun, Feixue Wang, Vadim Makarov

**Affiliations:** College of Electronic Science and Technology, National University of Defense Technology, Changsha 410073, China; College of Computer Science and Technology, National University of Defense Technology, Changsha 410073, China; College of Computer Science and Technology, National University of Defense Technology, Changsha 410073, China; College of Computer Science and Technology, National University of Defense Technology, Changsha 410073, China; College of Computer Science and Technology, National University of Defense Technology, Changsha 410073, China; College of Computer Science and Technology, National University of Defense Technology, Changsha 410073, China; College of Computer Science and Technology, National University of Defense Technology, Changsha 410073, China; College of Computer Science and Technology, National University of Defense Technology, Changsha 410073, China; College of Computer Science and Technology, National University of Defense Technology, Changsha 410073, China; College of Electronic Science and Technology, National University of Defense Technology, Changsha 410073, China; College of Electronic Science and Technology, National University of Defense Technology, Changsha 410073, China; Russian Quantum Center, Skolkovo, Moscow 121205, Russia; Vigo Quantum Communication Center, University of Vigo, Vigo E-36310, Spain; NTI Center for Quantum Communications, National University of Science and Technology MISiS, Moscow 119049, Russia

**Keywords:** quantum key distribution, penetration testing, black box

## Abstract

Quantum key distribution (QKD) establishes a shared secret between remote parties and is proven unbreakable in theory. Unfortunately, practical implementations of QKD exhibit device imperfections that lead to security vulnerabilities. Most of these vulnerabilities have been verified in a white-box testing scenario, when one has access to the system hardware for analysis. Here we implement automated penetration testing of a QKD system in a black-box setting, using only its public communication lines and a limited operator’s manual. Our implementation parses information transmitted over the classical communication line and toggles an optical delay in the quantum communication line. This enables it to tamper with the timing settings of detector gates in the QKD system during its calibration procedure and to passively eavesdrop on 98.97% of the sifted key. The entire testing process is fully automated and takes only minutes to initiate eavesdropping. Our work paves the way for automated penetration testing of QKD installations as a method of security verification.

## Teaser

Automated penetration test of QKD system as a black-box manipulates detector timing during calibration to learn 98.97% of the sifted key within minutes.

## INTRODUCTION

Quantum key distribution (QKD) is a promising technology in quantum cryptography, allowing two parties to securely share a random secret key through a public channel whose security is guaranteed by the principles of quantum physics [1]. Technically, commercial QKD systems are available [2–8], and advanced QKD implementations reach distances of up to $1002\, \mathrm{k}\mathrm{m}$ [9]. Although the QKD protocol provides information-theoretic security in principle [10], deviations of hardware from the ideal model in practice remain a significant problem. Various attacks exploit imperfections in components of QKD systems, such as photon sources [11–15], quantum-state modulators [12,16–19] and detectors [20–27], to eavesdrop on secure keys. So far, all known attacks are white-box attacks, requiring the adversary to know the internal details of the QKD system and to finely tune complex parameters to execute a successful attack [21,28,29]. Certification methodologies for QKD are also built around the white-box scenario [30,31]. Meanwhile, penetration testing with limited or no knowledge of the target system is standard in the security industry [32,33]. This demand is becoming more urgent as quantum communication moves towards scalable, networked architectures [34,35].

In this work, we propose and implement a plug-and-play attack on a decoy-state BB84 [36–38] QKD system treated as a black box. That is, Eve is permitted to launch attacks only via the quantum and classical channels, with no access to the internal components of the QKD system. Instead, she obtains only public information about the tested QKD system from the user manual and software interface, such as the protocol employed, repetition frequency, optical wavelength and system operating process, and has no prior knowledge of the existence of any vulnerability. By analyzing public documentation and the classical information exchanged between Alice and Bob, we identify a vulnerability during a specific calibration procedure, in which Eve actively induces a timing mismatch between the gate positions of paired single-photon detectors (SPDs) within a measurement basis [39–41]. Subsequently, in the raw-key exchange phase, Eve exploits this mismatch to eavesdrop on the secret key by continuously manipulating the arrival time of quantum states. Notably, this attack is automated and operates independently of the encoding degrees of freedom used in the QKD system under test. Moreover, it manipulates the transmission time and path of quantum states without requiring any intercept-resend operations [42,43].

Based on this security vulnerability, we implement a plug-and-play hacking (PPH) apparatus under realistic conditions over the channels between Alice and Bob. Our apparatus comprises both hardware and software components. Its hardware enables Eve to adjust the length of the quantum channel and to wiretap information from the classical channel. Meanwhile, the software interprets all data on the classical channel and dynamically tunes attack parameters in real time based on the status of the QKD system under test. Through this process, it successfully obtains Bob’s basis choices and calculates his partial quantum bit error rate (QBER) in both bases. By correlating this information with the length switch between short and long paths, Eve deduces Bob’s sifted key. The capability to automatically execute attacks and optimize hacking parameters within minutes is demonstrated over multiple QKD sessions lasting a few hours, successfully eavesdropping on 98.97% of the sifted key.

## RESULTS

### System under test

The engineering-validated QKD prototype served as the black-box system under test. From the public documentation, it is known that this QKD system features four detectors and employs a decoy-state BB84 QKD protocol with polarization coding and passive basis selection. The system, integrating both fully functional hardware and software, operates automatically to ensure reliable generation of secure keys.

**Figure 1.**
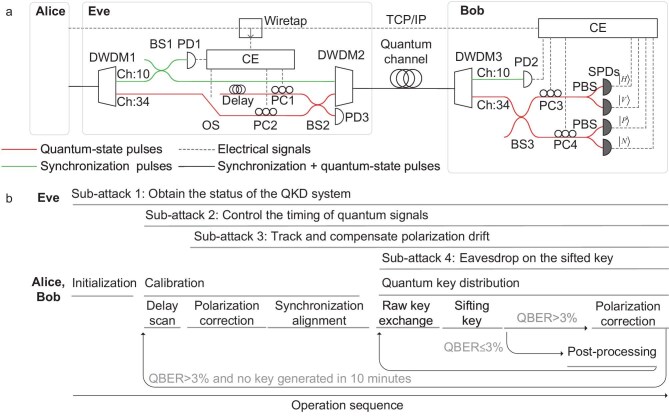
Plug-and-play hacking experiment. (a) Scheme of Eve and the black-box QKD system. Alice and Bob are black boxes, whereas Bob’s internal structure is shown as a speculative diagram. DWDM, dense-wavelength-division multiplexer; BS, beam splitter; PBS, polarization beam splitter; OS, optical switch; PD, photoelectric detector; SPD, single-photon detector; CE, control electronics; PC, programmable polarization controller; Ch, wavelength channel; $|H \rangle$, $|V \rangle$, $|P \rangle$, $|N \rangle$, SPD output signals corresponding to the four polarization states. (b) Timing of the four subattacks in the PPH apparatus.

Figure 1a depicts a schematic of the hardware in the QKD system. Alice transmits quantum-state pulses at a repetition frequency of 40 $\mathrm{M}\mathrm{Hz}$, which are sent over a single-mode fiber together with synchronization pulses using a dense wavelength-division multiplexing (DWDM) technique. After receiving the combined optical signals from Alice, Bob uses his DWDM3 module to demultiplex the synchronization pulses and quantum-state pulses. The former are detected by a photodetector (PD2) to synchronize Bob’s clock with Alice’s. The quantum signals are split into two equal parts via a 50:50 beam splitter (BS3). Each part undergoes polarization correction before being directed through a polarization beam splitter (PBS), which separates orthogonally polarized quantum states that are then detected by two SPDs.

The software operation sequence of the QKD system is shown in Fig. 1b, as observed from the user interface. Its has three stages: initialization, calibration and quantum key distribution. During initialization, the software is started and the connection between Alice and Bob is established. Calibration involves three phases. First is delay scanning, in which Alice sends four polarization states, namely $|H \rangle$ (horizontal), $|P \rangle$ ($45^\circ$), $|V \rangle$ (vertical) and $|N \rangle$ ($135^\circ$). Bob then determines each SPD’s gate position by detecting the maximum number of counts.

Second is polarization correction. Alice initially transmits $|H \rangle$ states, and Bob adjusts his polarization controller (PC3) until the click ratio $|H \rangle$: $|V \rangle$ reaches 99:1. Then, Alice sends $|P \rangle$ states, and Bob adjusts PC4 using the same procedure to achieve a similar click ratio. Third is synchronization alignment, in which Bob locks his clock to Alice’s using synchronization pulses.

At the quantum key distribution stage, Alice randomly sends four polarization states $|H \rangle$, $|V\rangle$, $|P \rangle$ and $|N \rangle$. Bob randomly chooses either the *X* or *Z* basis for detection via passive basis selection. Alice and Bob repeat this process to accumulate a sufficiently long raw key over a single raw-key exchange period. They then announce the basis of each click event to obtain the sifted key and reveal a small portion of the sifted key to calculate the QBER. If the QBER is less than 3%, the system performs post-processing, such as error correction and privacy amplification. Otherwise, the system repeats polarization correction in an attempt to reduce the QBER. If no key is generated within 10 minutes, the QKD system restarts from the beginning of the calibration stage.

### Vulnerabilities of a black-box QKD system

Eve wiretaps the classical channel to intercept communications between the transmitter, Alice and the receiver, Bob. By analyzing the public documentation and the classical information exchanged between Alice and Bob, we identify a vulnerability during the calibration procedure. We infer that Eve can actively modify detector gate positions and synchronization parameters only by hacking the QKD system through both the quantum channel and classical channels. Her detailed workflow is as follows.

In the QKD system, Bob independently adjusts each SPD’s gate position to achieve optimal detection efficiency during the calibration procedure. This process allows Eve to manipulate the arrival time of quantum states at Bob by selecting a long or a short path in the quantum channel, thereby independently controlling the gate position of each SPD [41]. As a result, a mismatch between the gate positions of paired SPDs within a measurement basis is actively created by Eve. During the raw-key exchange process, Eve can then exploit this mismatch to learn the secret-key information by continuously controlling the arrival time of quantum states at Bob, as she does during calibration.

### Implementation of a plug-and-play attack

Eve’s PPH apparatus (Fig. 1a) is independent of the encoding degrees of freedom of the system under test. It controls both the quantum and classical channels. Eve separates Alice’s signal into a $1550.12\,\mathrm{n}\mathrm{m}$ (wavelength channel 34) quantum signal and a $1569.59\, \mathrm{n}\mathrm{m}$ (wavelength channel 10) synchronization signal via DWDM1. The synchronization signal is then split by BS1: one part enters DWDM2, and the other part is detected by PD1, which generates a trigger signal for the control electronics (CE). The latter route the quantum signal through an optical switch (OS) to either a long or short path, thereby controlling its arrival time at Bob. Both paths are equipped with programmable PCs to compensate for polarization drift. The quantum-state pulses and synchronization pulses are subsequently recombined via BS2 and DWDM2 before being sent to Bob. Eve’s apparatus operates automatically throughout the entire QKD process, executing four distinct sub-attacks at different stages, as shown in Fig. 1b. The specific procedure of each sub-attack is as follows.

Sub-attack 1 begins by monitoring the information transmitted by the QKD system on the public channel from the authentication stage. Specifically, Eve wiretaps the classical channel to decode data and obtain the system status in real time, extracting operational parameters for subsequent attacks. This sub-attack enables the PPH apparatus to operate without requiring an in-depth understanding of the internal structure of the QKD system. In other words, it does not require comprehensive knowledge of the internal implementation details. Instead, as long as the basic communication protocols and interfaces are understood, the PPH apparatus can be inserted into the system to launch an attack. It should be noted that the tested QKD system encrypts classical communication using a private protocol. However, Eve has conducted reverse engineering at the algorithm level, which is purely classical software work and is not presented in detail here.

**Figure 2.**
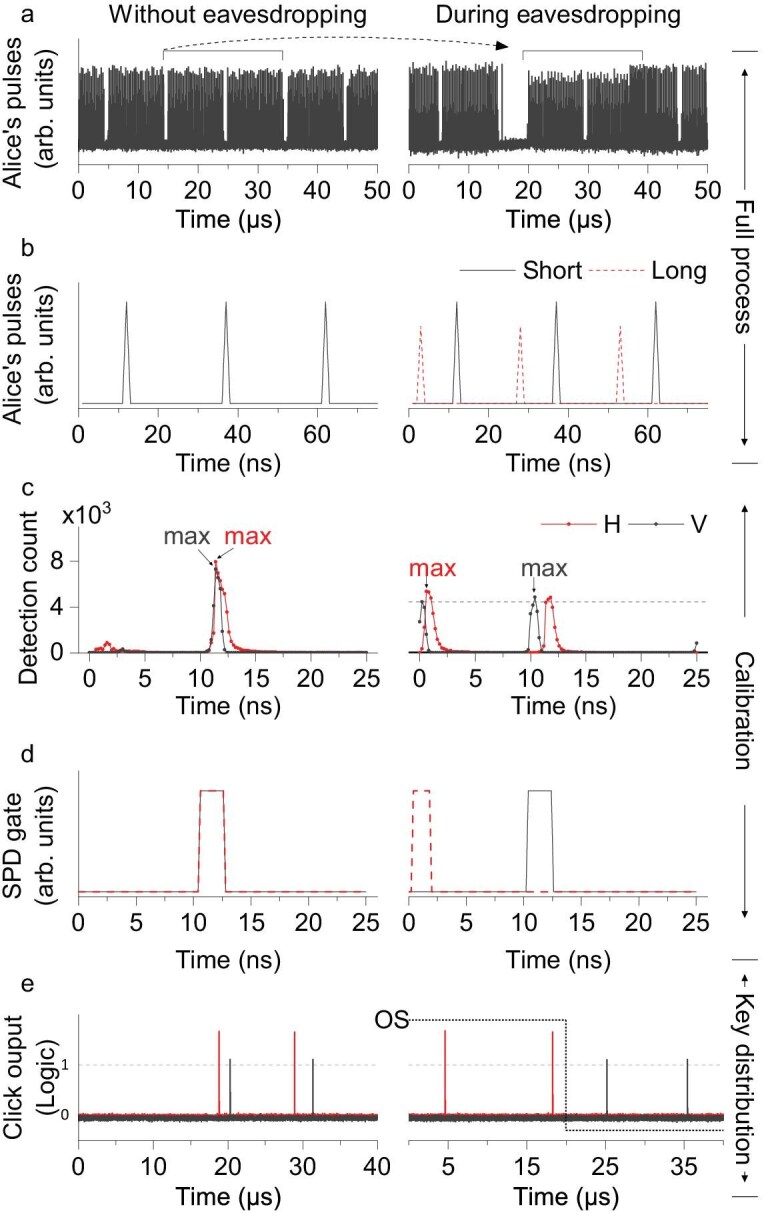
Experimental data from the QKD system. (a) Optical pulses emitted from Alice’s monitor port are detected by PD3 during the delay-scanning phase. (b) Timing sequences of signals detected by SPDs under various transmission paths. (c) Counts detected at different gate positions of the SPDs. (d) Gate positions of the SPDs. (e) Detection signals during key distribution. The switching period of the OS is indicated by a dotted line.

Sub-attack 2 manipulates the arrival time of quantum signals starting from the delay-scanning phase, causing inconsistent gate positions for paired SPDs within the same basis. In this sub-attack, the timing at which Eve switches paths depends on the synchronization period of the tested system. Specifically, she taps the synchronization light via PD1, which triggers her CE to generate an electrical pulse sequence with a 40 $\mathrm{\mu }\mathrm{s}$ period (this value is correlated with the synchronization rate of the tested QKD system) and a 50% duty cycle to control the OS. The OS forces quantum states to pass through the short path during the first half of each 40 $\mathrm{\mu }\mathrm{s}$ period and the long path during the second half. This operation ensures that each of Bob’s SPDs exhibits two highly comparable peaks within a 25 $\mathrm{n}\mathrm{s}$ period. To visually demonstrate the effect of the PPH apparatus on Alice’s quantum-state pulses, unattenuated light emitted from the monitor port of Alice’s module is connected to the PPH apparatus and detected by PD3. Figure 2a illustrates that, without eavesdropping, the quantum-state pulses exhibit a uniform periodic pattern, whereas under eavesdropping the quantum-state pulses shift. Notably, owing to the synchronization-alignment stage, the QKD system maintains clock synchronization between Alice and Bob even under this significant shift. In the following test, Eve shifts the quantum-state pulses only within a 25 $\mathrm{n}\mathrm{s}$ period to introduce as little loss as possible. Figure 2b provides an enlarged view, showing that, when quantum-state pulses travel exclusively through the long path, only one pulse is detected within the 25 $\mathrm{n}\mathrm{s}$ period. By contrast, when some pulses travel through the long path while others take the short path, two pulses, separated by about 10 $\mathrm{n}\mathrm{s}$, are statistically detected within the same period.

During the delay scanning phase, Bob divides one signal period into 125 detection intervals of $0.2\, \mathrm{n}\mathrm{s}$ and determines the gate positions based on peak detection counts. Without eavesdropping (Fig. 2c and d, left), the peak counts of both SPDs for $| H \rangle$ and $| V \rangle$ are centered near the 11th nanosecond. During eavesdropping (Fig. 2c and d, right), however, the maximum count of the SPD for $| H \rangle$ shifts to the first nanosecond, while that of the SPD for $| V \rangle$ remains at the original position. (When the maximum counts of two detection peaks are comparable for each SPD, one of these peaks is then randomly selected. If Eve fails to separate the gate positions, she disconnects the channel, forcing Bob to restart calibration). This attack causes a mismatch in the gate positions of the paired SPDs in the *Z* basis; a similar effect occurs in the *X* basis. Notably, this mismatch does not trigger any alerts from the QKD system. Once the gate positions are set during calibration, they remain fixed. This fixed configuration ensures that, during the raw-key exchange stage, the four SPDs can respond promptly and accurately upon receiving the corresponding quantum states. As shown on the left side of Fig. 2e, both SPDs for the $| H \rangle$ and $| V \rangle$ states may click throughout the entire 40 $\mathrm{\mu }\mathrm{s}$ period. However, in the presence of eavesdropping, the timing distribution of the responses is altered, as shown on the right side of Fig. 2e. In this scenario, the $| H \rangle$ state is detected exclusively during the first half of the 40 $\mathrm{\mu }\mathrm{s}$ switching period, while the $| V \rangle$ state is detected solely during the second half. This occurs because, during the first half of the period, the SPD for $| H \rangle$ is active owing to alignment between the gate position and the quantum-state arrival time, whereas the SPD for $| V \rangle$ remains inactive owing to misalignment of its gate position; the situation is reversed during the second half of the period, as the OS switches approximately every $20\, \mathrm{\mu }\mathrm{s}$. This phenomenon demonstrates that the PPH apparatus is independent of the encoding degree of freedom in the QKD system and can be adapted to other BB84 QKD systems by fine-tuning the attack parameters to match the specific target system.

Sub-attack 3 corrects polarization drift starting from the polarization-correction phase. The transmission of quantum states through the long and short paths in sub-attack 2 can be characterized by the linear operators $\hat{E}_l$ (long path) and $\hat{E}_s$ (short path), respectively. For any input polarization state $|\psi \rangle \in \lbrace |H \rangle , |V \rangle , |P \rangle , |N \rangle \rbrace$, the output states after transmission are given by


(1) \begin{eqnarray*}
\hat{E}_l |\psi \rangle &=& \sqrt{1 - \epsilon } |\psi \rangle + e^{ i\theta } \sqrt{\epsilon } |\psi _\perp \rangle ,\\
\hat{E}_s |\psi \rangle &=& \sqrt{1 - \xi } |\psi \rangle + e^{ i\theta ^{\prime } } \sqrt{\xi } |\psi _\perp \rangle ,
\end{eqnarray*}

where $|\psi _\perp \rangle$ denotes the polarization state orthogonal to $|\psi \rangle$, $\epsilon$ and $\xi$ are the probabilities of polarization drift, and $\theta$ and $\theta ^{\prime }$ are the phase offsets associated with the drift components. In ideal polarization correction for a single path, Bob can compensate for such drift using his PCs in each measurement basis (Fig. 1a). However, the path-dependent drifts in sub-attack 2 result in $\hat{E}_l \ne \hat{E}_s$. To mitigate this inconsistency, Eve deploys PCs in both the long and short paths. She iteratively adjusts these PCs using real-time QBER feedback from Bob to align the drift operators such that $\epsilon \approx \xi$ and $\theta \approx \theta ^{\prime }$, keeping the QBER $< 3\%$ under the attack. This process begins during polarization correction and continues until system termination. It is worth noting that a fitting algorithm enables the PPH apparatus to compensate for the polarization-drift difference caused by quantum-state transmission through the long and short paths within minutes, thereby better concealing its presence. Additionally, it can adaptively correct polarization drift caused by environmental changes in real time. As a result, the eavesdropping attack becomes plug-and-play. Further details are given in the Methods section below.

Sub-attack 4 infers the sifted key by cross-referencing information from sub-attacks 1 and 2 during key distribution. To deduce the sifted key, Eve must satisfy two additional conditions beyond the mismatch between the gate positions of paired SPDs. First, she must synchronize her clock with Bob’s to determine which path the photon takes corresponding to which SPD of Bob clicks in the announced basis. Second, she must be certain the bit-value mapping of Bob’s SPDs. Through sub-attack 1, Eve obtains all the data transmitted by Alice and Bob over the classical channel, enabling her to meet the above two conditions. Specifically, using the public information from the first raw-key exchange period—namely, the detection slots and his corresponding basis choices announced by Bob, together with the sifted slots and the portion of the sifted key bits revealed by Alice—Eve aligns Bob’s detection events with the 40 $\mathrm{\mu }\mathrm{s}$ switching cycle of the OS. By further analyzing the bit values and the QBER disclosed by Alice and Bob, Eve deduces the bit-value mapping corresponding to Bob’s four SPDs. Consequently, Eve exploits these timing and mapping relationships to infer the sifted key in each subsequent key-distribution round. In our experiment, Eve successfully deduced 98.97% of the sifted key generated by the QKD system. Detailed calculations and analyses are given in the Methods section below.

These above-mentioned sub-attacks work in concert, enabling Eve to remotely deploy the PPH apparatus and eavesdrop on the secret key. It should be noted that, since the PPH apparatus wiretaps information from the classical channel and modifies the length of the public quantum channel, it neither breaks the security assumptions of the BB84 QKD protocol nor violates the principles of quantum physics. As a result, it remains undetected by the QKD system.

### Attack performance

**Figure 3.**
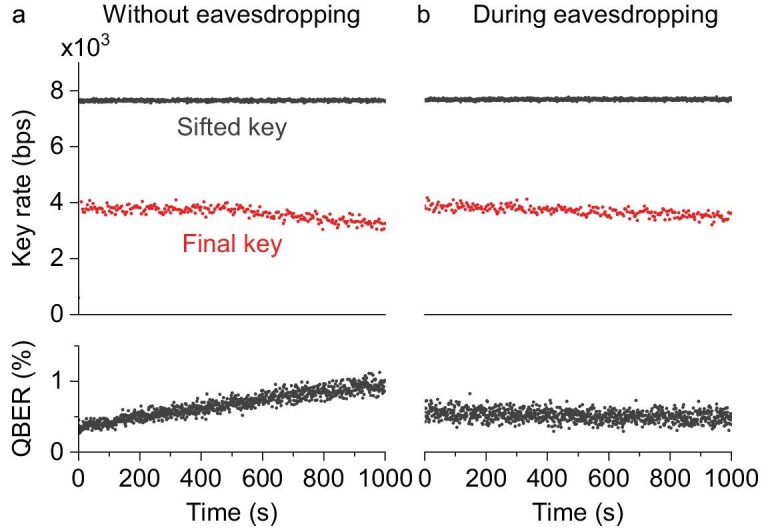
QKD performance with and without eavesdropping as measured by Alice and Bob. (a) Sessions without Eve in the fiber-optic line. (b) Sessions in which Eve eavesdrops on the fiber-optic line. The traces in the graphs, from top to bottom, correspond to the sifted key rate, the final key rate after error correction and privacy amplification, and the QBER.

We conducted multiple 10–$30\, \mathrm{min}$ QKD sessions over a few hours, with and without the attack. During these sessions, we recorded performance statistics, all public communication data exchanged between Alice and Bob, and the key-generation rate. In the key-distribution stage, the legitimate parties continuously monitored the key rates and QBER to assess the security of the transmission line. Figure 3 presents results from two representative sessions—one with eavesdropping by Eve and one without. These results show that Eve’s attack does not substantially affect the key rates. This occurs because the attack introduces no detectable QBER fluctuations that would alert Alice and Bob to Eve’s presence. The slight increase in the QBER without eavesdropping is caused by normal polarization drift during operation of this QKD system. However, during eavesdropping sessions, the QBER becomes more stable owing to the PPH apparatus’s real-time polarization compensation of environmental changes. Consequently, the QBER remains within the acceptable range ($<\!\! 3\%$) in both cases. Since Eve obtains almost the same sifted key as Bob, she can, in principle, apply post-processing similar to that of Alice and Bob and generate the same final key.

## DISCUSSION AND CONCLUSION

In this work, we demonstrate that, during calibration, Eve can induce a mismatch in the gate positions of Bob’s SPDs by controlling the different arrival times of quantum states at Bob. This makes our proposed attack independent of the encoding degree of freedom. As a result, the countermeasure of eliminating SPD gate mismatches through randomization of basis choices during calibration [41] is not applicable to this attack.

The QKD system needs to be equipped with an intrinsic mechanism as a countermeasure to eliminate this vulnerability. A free-running SPD may be an alternative, but its effectiveness against this calibration attack remains to be verified in future work. For example, a key consideration is how to determine the effective counting time window of a free-running SPD. An alternative approach could involve a time-correlated randomness test module into the QKD system [44,45], thereby enhancing the system’s ability to detect Eve’s presence. Notably, to counter this attack, Bob can also employ a ‘four-state measurement’ scheme [31,39]. In this scheme, he randomly selects both his measurement basis and the mapping of each SPD outcome to the logical bits 0 and 1. During the sifting process, Bob can deduce Alice’s encoded information by combining his own SPD results, his chosen states and the bases announced by Alice. The mapping of each SPD may be flipped, but it remains private and is never disclosed publicly. As a result, even if Eve knows which SPDs click, she cannot infer the actual bit value. However, this scheme still has a potential loophole. For example, Eve may attempt to inject a strong pulse to read out Bob’s detector assignments, similar to the strategy employed in a Trojan-horse attack [17,46,47]. More importantly, we recommend that QKD systems employ the measurement-device-independent QKD protocol [48] and its derivatives, such as twin-field QKD [49–51], sending-or-not-sending twin-field QKD [52] and side-channel-secure QKD [53,54]. In these protocols, the entire measurement unit is treated as an untrusted third party, Charlie. As a result, they theoretically guarantee the security of a QKD system, regardless of Eve’s attack on the detection side.

To summarize, we have successfully implemented a penetration testing on a QKD system as a black box through an online plug-and-play attack. This attack allows Eve to manipulate gate positions and synchronization parameters without access to the internal components of the QKD engine, thereby learning the secret-key information. Notably, this vulnerability and attack exploit the fact that the gate position of each SPD is calibrated separately. This means that this attack should be independent of the encoding degree of freedom, making it potentially applicable to other BB84-based QKD systems. Furthermore, this plug-and-play attack provides a testbed for online penetration testing, advancing methodology and techniques for the security certification of QKD.

## METHODS

### Sub-attack 3

The goal of compensating polarization drift is to minimize the polarization difference between the long path and short paths, thereby obtaining similar QBERs for both quantum states in the *Z* and *X* bases. Thus, the objective of sub-attack 3 is to compensate for the polarization difference between the long and short paths, that is, to achieve $\epsilon \approx \xi$ and $\theta \approx \theta ^{\prime }$, by reducing the deviation among the QBERs for different quantum states $\Delta _{error}$,


(2) \begin{eqnarray*}
\Delta _{error} = | \Delta _{H} - \Delta _{V} | + |\Delta _{P}-\Delta _{N}|,
\end{eqnarray*}

where $\Delta _{H}$, $\Delta _{V}$, $\Delta _{P}$ and $\Delta _{N}$ represent the QBERs of the signal states corresponding to $| H \rangle$, $| V \rangle$, $| P \rangle$ and $| N \rangle$, respectively. This QBER information is obtained from sub-attack 1.

To minimize the QBERs, Eve controls her PCs by adjusting the angles of polarization rotation along the *X, Y* and *Z* axes. Here, we fit the data using a curve function and set the values of the *X, Y* and *Z* axes to minimize $\Delta _{{error}}$. Sub-attack 3 starts during polarization correction and remains active throughout the QKD process, continually refining its estimations. As data accumulate, the attack adjusts the polarization-offset corrections more accurately. Consequently, the system’s QBER stabilizes at a value below 3%.

### Sub-attack 4

In sub-attack 4, Eve selects the first raw-key exchange period to establish synchronization and correlations among the basis, bit values and SPD clicks. She is then able to obtain the sifted key shared between Alice and Bob in subsequent raw-key exchange periods based on these correlations. Subsequently, Eve applies error correction and privacy amplification to obtain the final key, which is identical to that shared between Alice and Bob. The following describes how Eve obtains the sifted key.

**Figure 4.**
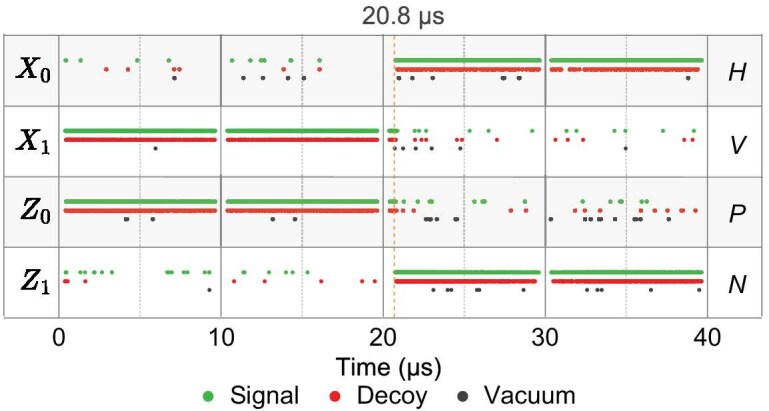
Alice publicly announces a portion of the valid click events and bit values at the first raw-key exchange period.

At the end of each raw-key exchange period, Bob communicates to Alice the details of all valid click events, including the chosen basis and the precise timing slots of these clicks. Subsequently, Alice publicly announces the instances in which she and Bob used the same basis at corresponding time sequences; this procedure is known as the sifting process in QKD systems. At the same time, Alice also publicly reveals a small subset of the sifted key bits so that Bob can calculate the QBER. Following this, Bob provides Alice with detailed QBER information, including the total number of valid events and the number of errors for each quantum state. In the PPH apparatus, the above transmission data are wiretapped by sub-attack 1. Sub-attack 4 then divides all valid click events within a raw-key exchange period into units of a 40 $\mathrm{\mu }\mathrm{s}$ switching period, as shown in Fig. 4. It can be seen that the majority of response events in the initial 20.8 $\mathrm{\mu }\mathrm{s}$ interval originate from quantum states corresponding to $| V \rangle$ and $| P \rangle$, whereas responses in the subsequent 19.2 $\mathrm{\mu }\mathrm{s}$ interval are attributed to $| H \rangle$ and $| N \rangle$ quantum states. Therefore, Eve can synchronize her clock with Bob’s by leveraging the timing distribution of Bob’s SPD click events, without resorting to complex physical means such as time-to-digital converter devices.

After Bob has completed the basis comparison with Alice, he compares his own data with the data that Alice has publicly disclosed. He then shares part of the sifted key with Alice to calculate the QBER, as illustrated in Table 1; this subset of the sifted key is subsequently discarded during post-processing. Similarly, this QBER information is wiretapped by sub-attack 1 in the PPH apparatus. Sub-attack 4 then analyzes the error counts and the total numbers for each quantum state in the QBER information. Despite the imbalance in counts between different measurement bases, Eve can mitigate it by increasing the OS modulation speed or introducing higher channel attenuation. Eve infers that the click events of $Z_0$, $Z_1$, $X_0$ and $X_1$ correspond to Bob’s $|H\rangle$, $|V\rangle$, $|P\rangle$ and $|N\rangle$ SPDs, respectively. The relationship between the bit values and the SPDs in each basis is shown on the right side of Fig. 4. Consequently, when Bob announces the commencement of the following raw-key exchange period, Eve uses the information from sub-attack 2 to determine whether Bob is responding to the $|H\rangle$/$|N\rangle$ states associated with the short path or the $|V\rangle$/$|P\rangle$ states associated with the long path at each click slot. Using this information, Eve reconstructs the sifted key shared between Alice and Bob.

**Table 1.** The information disclosed during the first round of QBER estimation.

	Signal state			Decoy state			
Bob’s SPD	Errors	Total	QBER (%)	Errors	Total	QBER (%)	Vacuum state
H	12	1042	1.15	11	809	1.36	14
V	6	2405	0.25	1	1039	0.01	7
P	5	2083	0.24	5	308	1.62	22
N	5	2245	0.22	6	871	0.69	12

**Figure 5.**
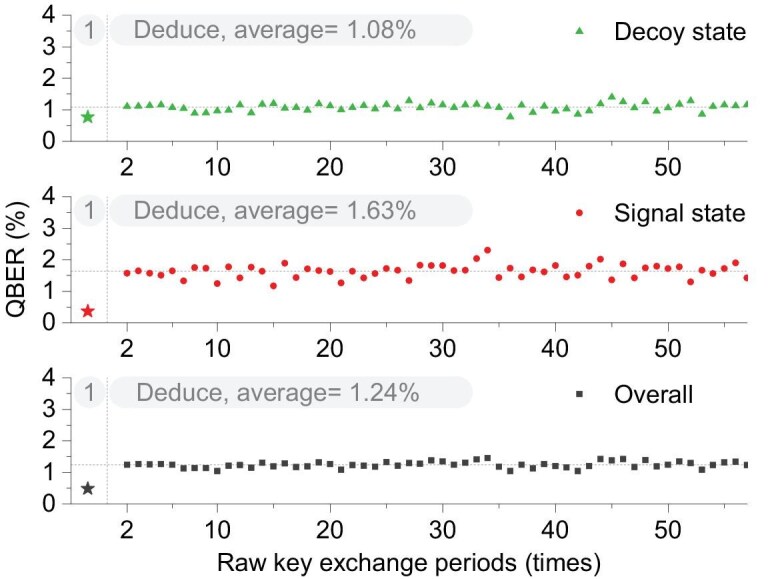
The QBER value calculated for 60 key-exchange periods. The first value represents the QBER between Alice and Bob and is marked with stars. The subsequent values represent the QBER between Alice and Eve.

Furthermore, to verify the accuracy of Eve’s sifted key, we compare Eve’s bit values with those disclosed by Alice and Bob to estimate the QBER in the next 60 rounds of the key exchange. Figure 5 shows the QBER of the signal states, decoy states and overall bit values between Eve and Alice. It can be seen that Eve’s average overall error rate is 1.24%, with a minimum of $1.03\%$. This enables Eve to apply error correction and privacy amplification to eavesdrop on the final key. It should be noted that, owing to factors such as optical-switch leakage, dark counts ($p_d$), polarization drift and other effects that cause Bob’s SPDs to click, Eve is unable to reconstruct some quantum-bit data for events beyond her control. For instance, the error rate for Eve is $e_0=p_d$, whereas for Bob it is $e_0=0.5\times p_d$.

## References

[1] Lo HK, Curty M, Tamaki K. Secure quantum key distribution. Nat Photon 2014; 8: 595–604. 10.1038/nphoton.2014.149

[2] ID Quantique . Quantum-Safe Security & Quantum Detection Systems. http://www.idquantique.com (16 March 2026, date last accessed).

[3] QuantumCTek . QuantumCTek-Quantum Secures Every Bit. http://www.quantum-info.com (16 March 2026, date last accessed).

[4] Qasky . Anhui ASKY quantum Technology CO., LTD. with a minimum http://www.qasky.com (16 March 2026, date last accessed).

[5] QRate . QRate: Quantum encryption. Security guaranteed by the laws of physics. https://goqrate.com (16 March 2026, date last accessed).

[6] Toshiba . Global Top Page. https://www.global.toshiba/ww/top.html (16 March 2026, date last accessed).

[7] LuxQuanta . Quantum Key Distribution - LuxQuanta. https://www.luxquanta.com (16 March 2026, date last accessed).

[8] QTI . QTI Quantum Telecommunications Italy. https://www.qticompany.com (16 March 2026, date last accessed).

[9] Liu Y, Zhang WJ, Jiang C et al. Experimental twin-field quantum key distribution over 1000 km fiber distance. Phys Rev Lett 2023; 130: 210801. 10.1103/PhysRevLett.130.210801 37295116

[10] Gottesman D, Lo HK, Lutkenhaus N et al. Security of quantum key distribution with imperfect devices. In: Proceedings of the International Symposium on Information Theory 2004.

[11] Nauerth S, Fürst M, Schmitt-Manderbach T et al. Information leakage via side channels in freespace BB84 quantum cryptography. New J Phys 2009; 11: 065001. 10.1088/1367-2630/11/6/065001

[12] Huang A, Navarrete Á, Sun SH et al. Laser-seeding attack in quantum key distribution. Phys Rev Appl 2019; 12: 064043. 10.1103/PhysRevApplied.12.064043

[13] Huang A, Li R, Egorov V et al. Laser-damage attack against optical attenuators in quantum key distribution. Phys Rev Appl 2020; 13: 034017. 10.1103/PhysRevApplied.13.034017

[14] Ponosova A, Ruzhitskaya D, Chaiwongkhot P et al. Protecting fiber-optic quantum key distribution sources against light-injection attacks. PRX Quantum 2022; 3: 040307. 10.1103/PRXQuantum.3.040307

[15] Peng Q, Chen JP, Xing T et al. Practical security of twin-field quantum key distribution with optical phase-locked loop under wavelength-switching attack. npj Quantum Inf. 2025; 11: 7. 10.1038/s41534-025-00963-9

[16] Gisin N, Fasel S, Kraus B et al. Trojan-horse attacks on quantum-key-distribution systems. Phys Rev A 2006; 73: 022320. 10.1103/PhysRevA.73.022320

[17] Jain N, Anisimova E, Khan I et al. Trojan-horse attacks threaten the security of practical quantum cryptography. New J Phys 2014; 16: 123030. 10.1088/1367-2630/16/12/123030

[18] Lu FY, Ye P, Wang ZH et al. Hacking measurement-device-independent quantum key distribution. Optica 2023; 10: 520–7. 10.1364/OPTICA.485389

[19] Ye P, Chen W, Zhang GW et al. Induced-photorefraction attack against quantum key distribution. Phys Rev Appl 2023; 19: 054052. 10.1103/PhysRevApplied.19.054052

[20] Kurtsiefer C, Zarda P, Mayer S et al. The breakdown flash of silicon avalanche photodiodes—back door for eavesdropper attacks? J Mod Opt 2001; 48: 2039–47. 10.1080/09500340108240905

[21] Lydersen L, Wiechers C, Wittmann C et al. Hacking commercial quantum cryptography systems by tailored bright illumination. Nat Photon 2010; 4: 686–9. 10.1038/nphoton.2010.214

[22] Bugge AN, Sauge S, Ghazali A et al. Laser damage helps the eavesdropper in quantum cryptography. Phys Rev Lett 2014; 112: 070503. 10.1103/PhysRevLett.112.070503 24579579

[23] Huang A, Sajeed S, Chaiwongkhot P et al. Testing random-detector-efficiency countermeasure in a commercial system reveals a breakable unrealistic assumption. IEEE J Quantum Electron 2016; 52: 8000211. 10.1109/JQE.2016.2611443

[24] Wu Z, Huang A, Chen H et al. Hacking single-photon avalanche detectors in quantum key distribution via pulse illumination. Opt Express 2020; 28: 25574–90. 10.1364/OE.397962 32907074

[25] Gao B, Wu Z, Shi W et al. Ability of strong-pulse illumination to hack self-differencing avalanche photodiode detectors in a high-speed quantum-key-distribution system. Phys Rev A 2022; 106: 033713. 10.1103/PhysRevA.106.033713

[26] Su J, Chen J, Lu F et al. Security analysis of orthogonal state attack on a high-speed quantum key distribution system. [preprint] arXiv: 2506.03718v3.

[27] Kang X, Chen JL, Wang ZH et al. Hacking high-speed quantum-key-distribution systems by tailoring the blinding pulse. Phys Rev Appl 2026; 25: 024053. 10.1103/nqz3-z1zb

[28] Gerhardt I, Liu Q, Lamas-Linares A et al. Full-field implementation of a perfect eavesdropper on a quantum cryptography system. Nat Commun 2011; 2: 349. 10.1038/ncomms1348 21673670

[29] Gisin N . Quantum cryptography: where do we stand? [preprint] arXiv: 1508.00341v1.

[30] ISO/IEC 23837-2:2023(en) . Information Security—Security Requirements, Test and Evaluation Methods for Quantum Key Distribution—Part 2: Evaluation and Testing Methods. https://www.iso.org/obp/ui/en/#iso:std:iso-iec:23837:-2:ed-1:v1:en (16 March 2026, date last accessed).

[31] Makarov V, Abrikosov A, Chaiwongkhot P et al. Preparing a commercial quantum key distribution system for certification against implementation loopholes. Phys Rev Appl 2024; 22: 044076. 10.1103/PhysRevApplied.22.044076

[32] Alhamed M, Rahman MMH. A systematic literature review on penetration testing in networks: future research directions. Appl Sci 2023; 13: 6986. 10.3390/app13126986

[33] Bacudio AG, Yuan X, Chu BTB et al. An overview of penetration testing. Int J Netw Secur Appl 2011; 3: 19–38.

[34] Lu FY, Wang ZH, Zhou Y et al. Fully heterogeneous prepare-and-measure quantum network for the next stage of quantum internet. Nat Commun 2025; 16: 11487. 10.1038/s41467-025-66333-3 41387686 PMC12749763

[35] Zheng Y, Wang H, Jia X et al. Large-scale quantum communication networks with integrated photonics. Nature 2026; 651: 68–75. 10.1038/s41586-026-10152-z 41673161 PMC12960210

[36] Wang XB . Beating the photon-number-splitting attack in practical quantum cryptography. Phys Rev Lett 2005; 94: 230503. 10.1103/PhysRevLett.94.230503 16090451

[37] Lo HK, Ma X, Chen K. Decoy state quantum key distribution. Phys Rev Lett 2005; 94: 230504. 10.1103/PhysRevLett.94.230504 16090452

[38] Ma X, Qi B, Zhao Y et al. Practical decoy state for quantum key distribution. Phys Rev A 2005; 72: 012326. 10.1103/PhysRevA.72.012326

[39] Qi B, Fung CHF, Lo HK et al. Time-shift attack in practical quantum cryptosystems. Quantum Inf Comput 2007; 7: 73–82.

[40] Zhao Y, Fung CHF, Qi B et al. Quantum hacking: experimental demonstration of time-shift attack against practical quantum-key-distribution systems. Phys Rev A 2008; 78: 042333. 10.1103/PhysRevA.78.042333

[41] Jain N, Wittmann C, Lydersen L et al. Device calibration impacts security of quantum key distribution. Phys Rev Lett 2011; 107: 110501. 10.1103/PhysRevLett.107.110501 22026652

[42] Bennett CH, Brassard G. Quantum cryptography: public key distribution and coin tossing. Theor Comput Sci 2014; 560: 7–11. 10.1016/j.tcs.2014.05.025

[43] Bennett CH, Bessette F, Brassard G et al. Experimental quantum cryptography. J Cryptology 1992; 5: 3–28. 10.1007/BF00191318

[44] Wooltorton L, Brown P, Colbeck R. Tight analytic bound on the trade-off between device-independent randomness and nonlocality. Phys Rev Lett 2022; 129: 150403. 10.1103/PhysRevLett.129.150403 36269949

[45] Hangleiter D, Eisert J. Computational advantage of quantum random sampling. Rev Mod Phys 2023; 95: 035001. 10.1103/RevModPhys.95.035001

[46] Vakhitov A, Makarov V, Hjelme DR. Large pulse attack as a method of conventional optical eavesdropping in quantum cryptography. J Mod Opt 2001; 48: 2023–38. 10.1080/09500340108240904

[47] Sajeed S, Minshull C, Jain N et al. Invisible Trojan-horse attack. Sci Rep 2017; 7: 8403. 10.1038/s41598-017-08279-1 28827553 PMC5566943

[48] Lo HK, Curty M, Qi B. Measurement-device-independent quantum key distribution. Phys Rev Lett 2012; 108: 130503. 10.1103/PhysRevLett.108.130503 22540686

[49] Wang XB, Yu ZW, Hu XL. Twin-field quantum key distribution with large misalignment error. Phys Rev A 2018; 98: 062323. 10.1103/PhysRevA.98.062323

[50] Lucamarini M, Yuan ZL, Dynes JF et al. Overcoming the rate–distance limit of quantum key distribution without quantum repeaters. Nature 2018; 557: 400–3. 10.1038/s41586-018-0066-6 29720656

[51] Wang S, Yin ZQ, He DY et al. Twin-field quantum key distribution over 830-km fibre. Nat Photon 2022; 16: 154–61. 10.1038/s41566-021-00928-2

[52] Jiang C, Yu ZW, Hu XL et al. Unconditional security of sending or not sending twin-field quantum key distribution with finite pulses. Phys Rev Appl 2019; 12: 024061. 10.1103/PhysRevApplied.12.024061

[53] Wang XB, Hu XL, Yu ZW. Practical long-distance side-channel-free quantum key distribution. Phys Rev Appl 2019; 12: 054034. 10.1103/PhysRevApplied.12.054034

[54] Jiang C, Hu XL, Yu ZW et al. Side-channel security of practical quantum key distribution. Phys Rev Res 2024; 6: 013266. 10.1103/PhysRevResearch.6.013266

